# CT-Guided Percutaneous Cryoablation of Breast Cancer: A Single-Center Experience

**DOI:** 10.3390/cancers16132373

**Published:** 2024-06-28

**Authors:** Thomas J. Vogl, John Bielfeldt, Ulrich Kübler, Hamzah Adwan

**Affiliations:** 1 Clinic for Radiology and Nuclear Medicine, University Hospital Frankfurt, Goethe University, Theodor-Stern-Kai 7, 60590 Frankfurt, Germanyadwan.hamza97@gmail.com (H.A.); 2 Labor Praxisklinik, Weltenburger Str. 70, 81677 Munich, Germany

**Keywords:** interventional radiology, cryoablation, breast cancer

## Abstract

**Simple Summary:**

Liquid-nitrogen-based cryoablation presents a minimally invasive therapeutic option for small local cancer of the breast. The aim of this study was to investigate the efficacy and safety of cryoablation according to safety and efficacy. Therefore, this study reports on the outcome of 45 patients who were treated with liquid nitrogen-based cryoablation at our clinic.

**Abstract:**

This study shall retrospectively evaluate the efficacy and safety of liquid-nitrogen based CT-guided cryoablation (CA) as a minimal-invasive technique for the curative treatment of primary breast cancer. A total of 45 female patients with 56 tumors were treated by CT-guided CA in analgosedation as an outpatient procedure. We used a liquid-nitrogen based system with a single cryoprobe and performed two freeze cycles with an intermediate thawing. The mean tumor diameter was 1.6 ± 0.7 cm. Follow-up was conducted via contrast-enhanced MR images of the breast. No complications were observed in all 56 ablations. Initial complete ablation was achieved in 100% of cases. Four cases of local tumor progression were reported, resulting in a rate of 8.9%, and 6 cases of intramammary distant recurrence at a rate of 13.3%. The extramammary tumor progression was observed in 7 patients at a rate of 15.6%. The mean overall survival was 4.13 years (95% CI: 3.7–4.5). The mean overall progression-free survival was 2.5 years (95% CI: 1.8–3.2) and the mean local progression-free survival was 2.9 years (95% CI: 2.3–3.6). Cryoablation is a safe and effective treatment for primary breast cancer tumors, which can be performed in analgosedation and as an outpatient procedure. However, potential for improvement exists and further evidence is necessary.

## 1. Introduction

Temperatures below freezing were first used in 1851 to treat breast cancer [1]. James Arnott (1797–1883) had invented a waterproof cushion, in which a mixture of saline water was conveyed to freeze skin-penetrating breast cancers, mainly as a palliative analgesic therapy [2]. Since then, the use of cold temperatures as a medical treatment has significantly expanded. However, it was only with the use of liquid nitrogen (LN) in 1950 that cryotherapy could be widely applied as a treatment for malignancies of the eye [3], brain [4], liver, kidneys, and other locations [5]. 

Current cryoablation (CA) devices make use of the Joule-Thompson effect, which allows a freezing process due to argon gas expansion. The gas is stored under high pressure, and if allowed to expand, it induces a dramatical drop in temperature, enabling a cooling process [1,6]. On the other hand, there are devices that utilize LN to cool the probe [7]. These are low cost and easy to manage, requiring only a single probe for the freezing process [8]. However, argon gas cryoablation devices enable the usage of more than one probe [9]. 

Several treatment methods can be applied for breast cancer including surgery, radiotherapy and chemotherapy [10]. Local ablation offers several advantages, especially compared to surgical resection. The index tumor can be visualized during the procedure to ensure an adequate ablation margin. It is also a cost-effective and time-efficient option, with a notably better cosmetic outcome [11].

With breast cancer being the most common type of cancer globally [12], and especially with rates of early diagnosis increasing [13], the need for further adequate, effective and safe minimally invasive treatments is also increasing. Many studies on the use of local ablation techniques have been performed, showing that radiofrequency ablation (RFA), microwave ablation (MWA), and CA are effective therapies with few complications in the treatment of breast cancer [11,14]. However, most of the studies discussing the outcome of CA in breast cancer patients had small sample sizes and mainly used ultrasound-guided techniques. For example, a meta-analysis by Peek et al. [11] included ten CA studies with a total of 269 patients, who were mostly treated by ultrasound-guided CA. Furthermore, two German studies discussing this topic could be found [15,16]. Until now, local-ablative techniques for the treatment of breast cancer are not recommended or mentioned in either the European or the German guidelines [10,17].

Therefore, this study aims to further highlight the outcomes of patients with breast cancer undergoing liquid-nitrogen based CT-guided CA and provide more evidence supporting the utilization of it as a minimally invasive therapy.

## 2. Materials and Methods

### 2.1. Ethical Statement

Approval of the university hospital’s ethics committee was acquired (Approval code: 2023-1358).

### 2.2. Study Design

We retrospectively reviewed patients treated with CA in our institute from May 2019 to May 2023. The inclusion criteria for the ablation procedures were as follows: (1) Patient over 18 years of age, (2) with breast cancer, (3) tumor unresectable because of patient’s general condition or patient not willing to undergo surgery, (4) tumor smaller than 4 cm in diameter, (5) three or less tumors (6) tumor without skin infiltration (7) tumor clearly visible on computed tomography. Patients with any of the following conditions were not treated via CA: (1) local and/or systemic infection, (2) severe coagulation disorders, (3) allergy to local anesthetic, (4) hemodynamic or respiratory instability. In cases of multiple tumors, each tumor was treated on separate occasions.

### 2.3. Cryoablation Procedure

To plan the CA procedure the patients were placed in a 1.5-T or 3-T MRI systems (Siemens) to obtain axial T1- and T2-weighted images, as well as coronar T1-weighted MR images and diffusion-weighted images of the breast. Following this, contrast agent was injected to acquire a contrast-enhanced axial dynamic 3D-sequence of the breast. Additionally, the patients’ latest blood count and coagulation status were examined before the procedure.

The IceCure medical ProSense™ ablation system was used for the cryoablation procedures. This system uses LN circulating through the tip of the cryoprobe to freeze it and, consequently, cool the surrounding tissue to create an ice ball. An experienced consultant performed the ablation, with the assistance of a specialized medical technical radiology nurse under CT-guidance using the ProSense™ System. General anesthesia was not administered; instead, analgosedation with diazepam and piritramide was carried out, and the patient’s vital parameters were monitored via electrocardiography, blood pressure, oxygen saturation, and respiratory rate.

Beforehand, a planning CT-scan was performed to locate an appropriate incision point and plan the trajectory of the probe shaft. This ensured that the cryoprobe could be inserted along the long axis of the tumor, trying to ensure complete ablation of the total tumor volume. 

To provide coolant for the procedure a 2-L dewar with LN is inserted into the back of the system before the initiation of the CA. Subsequently, the sterile, single use cryoprobe was attached to the handle and tested by inserting the tip into saline solution until an ice ball formed. If the test was successful, the cryoprobe was inserted into the index tumor. Prior to this, a thorough disinfection of the incision area was performed to ensure sterile conditions, and a local anesthetic was applied to the skin and surrounding tissue at the target incision point. Following this, a small incision in the skin was made, and the cryoprobe was inserted through this incision and properly placed under CT guidance inside the index tumor, ensuring that the center of the cool zone matched the center of the tumor. CT guidance was chosen for the ablation procedure to enable an exact visualization of the cryoprobe, tumor as well as ice ball in all dimensions and to ensure a sufficient safety distance to adjacent structures. 

To prevent skin burns, the marked safety zone of the cryoprobe was placed deep enough not to be visible from the outside. The CA procedure was commenced with the first freeze cycle, followed by a thaw period and then the second freeze cycle. Throughout the freeze cycles, repetitive CT-images were obtained to ensure a sufficiently growing ice ball that extended beyond the tumors margins and to prevent the ice ball from growing too close to the skin or thoracic wall. If a tumor was located closely to the skin or thoracic wall, saline solution for hydrodissection was carefully injected between the tumor and the skin or thoracic wall. 

If the first freeze cycle exceeded 5 min, a dewar refill was necessary between the two freeze cycles. Once the system completed a full ablation cycle and the ice formation exceeded the tumor margins by 0.5 cm in all dimensions, the tip of the cryoprobe was heated by circulating warm nitrogen through the probe’s tip. Subsequently, the cryoprobe was detached from the ice ball and safely removed from the index tumor.

Following the ablation, the patients were monitored for a couple of hours to check for any complications, and to ensure that they received pain medication if post-interventional pain was reported. The patients were discharged on the same treatment day. 

### 2.4. Follow-Up

The first post-interventional images were taken during the 24 h post-ablation. The patients were placed in the MRI to obtain axial T1- and T2-weighted images, coronar T1-weighted, and diffusion-weighted MR images. To ensure that no residue tumor remained after the ablation, the patients were also injected with 0.2 mL per kilogram of bodyweight of gadolinium contrast agent for a contrast-enhanced axial dynamic 3D-sequence of the breast. If any residual contrast-enhanced tumor tissue was observed at the ablation margins in the first follow-up, incomplete ablation was assumed, and another CA was performed to ensure complete ablation of the index tumor. Subsequent breast MRI follow-ups were scheduled at 3, 6, 9, and 12 months, respectively. After the first year patients received a follow-up biannually. The patients underwent mainly CT scans for detecting extramammary tumor manifestations.

### 2.5. Data and Statistical Analysis

The patients’ data were analyzed with regard to the number of tumors; axial diameter of the index tumor, ice ball and ablation area; ablation area volume and volume reduction; duration of ablation; complications; complete ablation; local tumor progression (LTP); intramammary distant recurrence (IDR); extramammary tumor progression (ETP); overall survival (OS) and progression free survival (PFS). Initial complete ablation, defined as an ice formation encompassing the tumor with a safety margin of 0.5 cm around the tumor margins. LTP was defined, as previously mentioned by Ahmed et al. [18], as new tumor foci growth adjacent to the ablation zone margins if an initially complete ablation had been achieved. IDR was defined as new tumor growth within the breast but not directly bordering the initial ablation zone. Diagnosis of LTP or IDR was made based on MRI. Complications were also documented according to the CIRSE Classification system [19]. The OS was calculated from the date of the first ablation until death or the last follow-up. The overall PFS was calculated starting from the day of CA until the date of the first registered event of either LTP, IDR, ETP, death or last follow-up. The local PFS was calculated starting from the day of CA until the date of either LTP, IDR, death or last follow-up. Statistical analysis was performed with SPSS^®^ (Statistical Package for the Social Sciences, GradPack 27.0, IBM, Armonk, NY, USA). The Kaplan-Meier Method was used to calculate the OS and PFS.

## 3. Results

### 3.1. Patients

A total of 45 female patients (mean age 55.6 ± 12.5 years, range: 31.3–86.0 years) who underwent CA treatment for breast cancer were included. A total of 56 procedures were performed on 56 tumors, in a curative intention for non-metastatic patients, as well as to achieve local tumor control in patients with metastases. Among them, nine patients had two tumors and one patient had three tumors, all treated on separate occasions. All patients were female. Consequently, one patient had two separate ablations due to tumor recurrence.

Of the 45 patients treated, 11 had tumors that were disease recurrences of preexisting tumors. Therefore, these patients had already received various therapies beforehand, such as surgery, radiation therapy, or chemotherapy. In Addition, 21 patients had a metastatic disease situation, with 85.7% (18/21) of these patients having axillary lymphatic metastasis. Patient and tumor characteristics can be seen in Table 1.

### 3.2. Index Tumor Size

The mean axial tumor diameter was 1.6 ± 0.7 cm (Range: 0.7–3.7 cm). Out of the tumors, 10 were above 2 cm in diameter (17.9%) and 46 were equal to or below 2 cm in diameter (82.1%). The mean volume of the tumors ablated was 2.3 ± 2.4 mL (Table 1).

### 3.3. Ablation Procedure

On average, the second ice ball was larger than the first one, with a mean length of 4.5 cm compared to 4.1 cm for the first ice ball, as the surrounding tissue is already cooled down sufficiently before the second freeze cycle, because of the failure of microcirculation after the initial freeze cycle [20]. The average ice ball length for both ablation cycles was 4.3 cm. The mean cumulative freezing time was 16.6 min.

Based on the first post-treatment MR images the average diameter of the ablation zone was 5.3 ± 1.4 cm, with an average volume of 38.6 ± 17.5 mL. Subsequent follow-up images showed an average reduction of 69.4 ± 16.5% in the ablation zone volume after 3 months. The reduction of the ablation site volume based on each of the last available follow-up MRI images was 80.5 ± 20.1%. 

**Figure 1:**
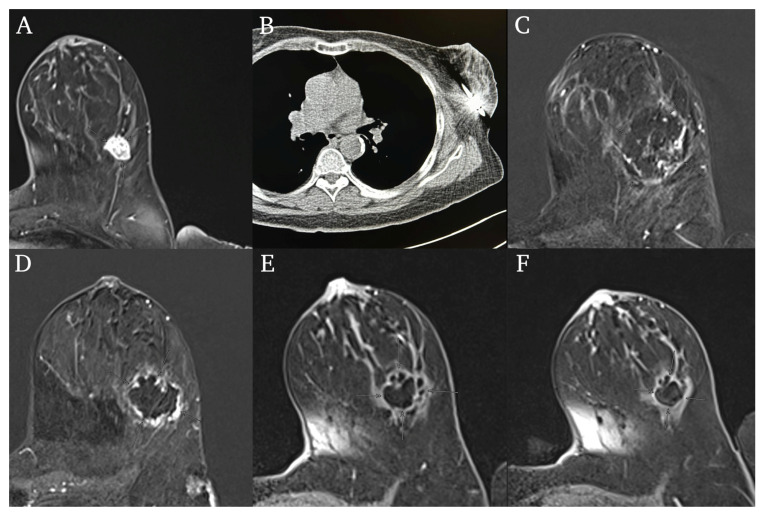
59-year-old female patient with cancer in the left breast. The Cryoablation was performed with two 9 min freeze cycles with a 9-min passive thaw cycle in between. (**A**) Pre-Ablation contrast-enhanced T1-weighted MR images. Tumor localized in left lower lateral quadrant. (**B**) The cryoprobe is inserted in the lesion during the CT-guided ablation procedure. (**C**) First post-ablation images, 24 h after ablation. The ablation zone can be seen in the contrast-enhanced T1-weighted MR images. (**D**) Ablation zone in the 3-month post follow-up MR-images. (**E**) Ablation zone in the 6-month post follow-up MR-images. (**F**) Ablation zone in the 9-month post follow-up MR-images. The patient showed a complete remission and had a survival time of 16 months starting at the date of ablation until last contact.

Initial complete ablation was reported in all ablations (56/56) based on the intra-procedural CT images and the first post-ablation MR images. Moreover, no complications or procedure-related deaths were reported. Henceforth, no complications regarding the CIRSE classification system were documented. The results from the ablation procedures can be seen in Table 2. An example of a patient’s case can be seen in Figure 1.

### 3.4. Outcome and Survival

Out of the 45 patients treated, we identified four cases of LTP, six cases of IDR and seven cases of ETP. Therefore, the LTP rate was 8.9%, the IDR rate was 13.3% and the ETP rate was 15.6%. One case of LTP, fives cases of IDR and one case of ETP were observed in patients without metastases. Three cases of LTP, one case of IDR and six cases of ETP were reported in patients with metastases. 

**Figure 2:**
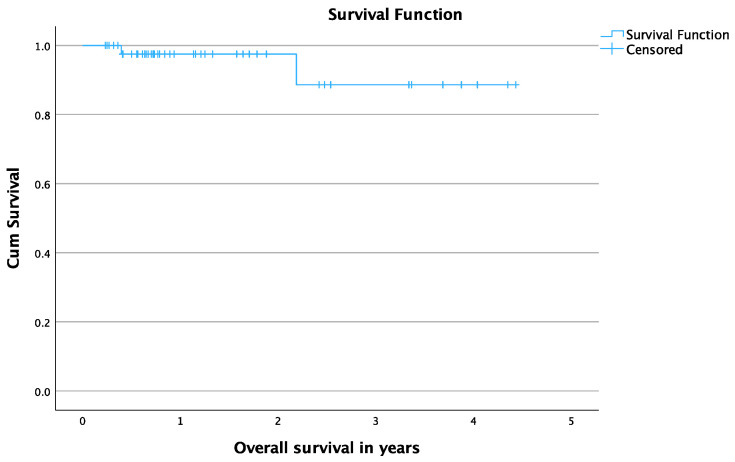
Overall survival in years for patients treated with Cryoablation.

The mean OS time was 4.13 years (95% CI: 3.7–4.5), as shown in Figure 2. The death of two patients was reported during the follow-up period due to ETP. 

**Figure 3:**
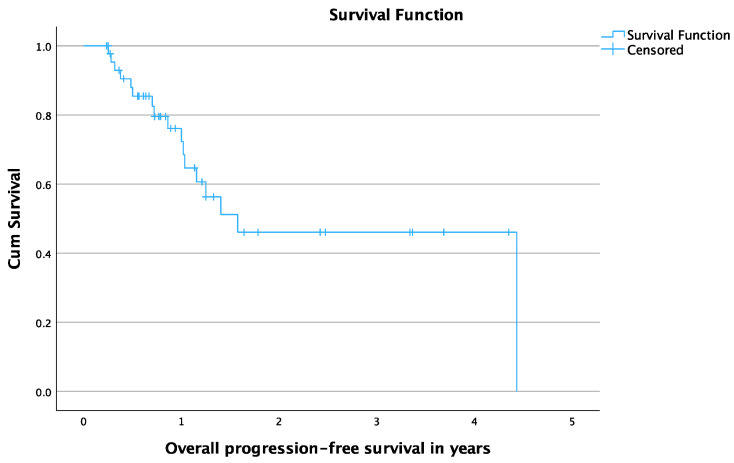
Overall progression-free survival in years for patients treated with Cryoablation.

**Figure 4:**
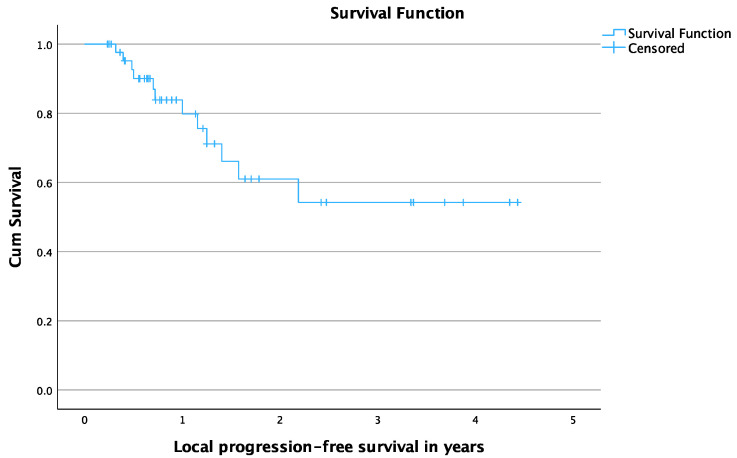
Local progression-free survival in years for patients treated with Cryoablation.

Additionally, the mean overall PFS time was 2.5 years (95% CI: 1.8–3.2) and the mean local PFS time was 2.9 years (95% CI: 2.3–3.6). The 6- and 12-month overall PFS rates were 85.4% and 72.3%, respectively. The 6- and 12-month local PFS rates were 90% and 80%, respectively. Figure 3 and Figure 4 show the Kaplan-Meier curves of overall PFS and local PFS, respectively. A summary of the patients’ outcome can be seen in Table 3.

## 4. Discussion

In this study, 45 patients with a total of 56 tumors were treated using CA. Many patients had received various therapies before CA, including surgery, radiation therapy, or chemotherapy. Additionally, 21 patients had a metastatic disease situation. The average OS time was 4.13 years. The mean overall PFS time was 2.5 years and the mean local PFS time was 2.9 years. LTP was observed in four cases and IDR in six cases. A total of seven patients had ETP.

With 71,000 new diagnoses and over 18,000 deaths per year breast cancer is a major health issue in Germany [21] and worldwide. After the implementation of a breast cancer mammography screening in Germany in 2005 the cases of ductal carcinoma in situ increased by 121%, with breast cancer incidence remaining high at 134 cases per 100,000 people [21]. UICC stage I and II account for the largest fraction of cases [21]. Statistically, one in eight women will receive a breast cancer diagnosis in her life [22], emphasizing the necessity for newer therapy options. While chemotherapy is well-established in breast cancer treatment [10], the mode of action is systemic and so side effects are also systemic. On the other hand, surgery does not include systemic side effects and enables axillary staging but requires general anesthesia and may not always achieve ideal cosmetic outcomes [23]. Therefore, minimal-invasive ablation techniques such as RFA, MWA and CA offer potential advantages, being time efficient and effective therapies with low complication rates for breast cancer [11]. These techniques can be repeated several times, do not require general anesthesia, and allow for shorter hospital stays and quicker recovery [11].

There are a few meta-analyses which have discussed the effectiveness and outcomes of minimal-invasive ablative techniques for breast cancer. For instance, in a meta-analysis by Peek et al. [11] CA had a complete ablation rate of 74.1%, compared to 100% in our study. Furthermore, they reported a local recurrence rate of 1.4%, whereas our study found an 8.9% rate of local recurrence. Additionally, 10.9% of patients in the CA studies included in the meta-analysis had complications, while our study reported no complications. On the other hand, CA had a relatively long treatment duration of 50.3 ± 58.4 min, compared to 15.6 ± 5.6 min with RFA and 19.0 ± 18.2 min with MWA. We did not keep track of the total treatment time in our study but registered an average combined freeze duration of 16.6 min. 

In nearly all studies found during a literature search, discussing CA of breast cancer, the index tumor was resected after the cryoablation procedure to evaluate the histopathological outcomes of cryoablation, and as a result, no PFS or OS times were calculated [15,24,25,26]. Only a study by Pusceddu et al. [27] on CA of stage IV metastatic breast cancer in 35 patients, recorded a mean time to local recurrence of 64.0 ± 4.7 months with a median follow-up of 46 months, and seven deaths (20%) due to tumor progression.

Furthermore, two prospective studies on CA of early stage breast cancer were found: In the 3-year interim analysis of the ICE 3 trial [28], which was funded by IceCure medical, the manufacturer of the ProSense CA device, it was reported that out of 194 patients with low-risk, early stage breast cancer treated with CA only 4 had an ipsilateral breast tumor recurrence, resulting in a recurrence rate of 2.4%, although the ablated tumors were much smaller (mean tumor length 0.8 cm) than those in our study (mean tumor length 1.6 cm). There is a second ongoing prospective study on this subject, the FROST trail (NCT01992250) [29], which is sponsored by Sanarus, a manufacturer of a different LN CA device, but no results have been published yet. 

In comparison to other studies where the complete ablation rate was up to 100% [30,31], our study had a similar complete ablation rate. However, the LTP rate in our study is worse than most studies. This could be explained by the fact that we did not only include low risk tumors smaller than 2 cm in diameter but tumors up to 4 cm in diameter and even patients with metastatic disease. 

Regarding the discussion whether argon-based CA devices or LN-based devices are better, it can be said that LN does not require specific security measures, as it is not stored in a pressurized container [9]. The mean length of the ice ball in our study with a single cryoprobe was 4.3 cm, while the mean ice ball size in the study by Littrup et al. [31] was 5.1 cm ± 2.2 cm, however with an average of 3.3 cryoprobes per tumor. In summary, LN based CA systems are cheaper and easier to use but also have some disadvantages in contrast to argon-based systems. 

Finally, our study has several limitations. It is a single center retrospective study, with a very heterogeneous study population, including many patients with recurrent tumors, and metastatic disease, most of the cases with axillary lymph node metastases. Therefore, the comparability of our study to others is limited, also due to the heterogeneity of the other studies on this subject. Secondly, this study was performed as a single-arm study, without a comparison to another ablation procedure or surgery. A prospective comparative study would provide further evidence. Finally, important factors such as the histopathological types of breast cancer were not investigated.

## 5. Conclusions

CA is a safe and effective local treatment for breast cancer, which can be performed in analgosedation and as an outpatient procedure. Complication rate is low and high complete ablation rate can be achieved. The patients had satisfactory OS, PFS as well as local tumor control, though improvement is possible especially towards lower LTP rates. Further evidence is still needed, as well as further studies with more homogenous group of patients, for a better comparability to other treatment modalities. 

## Figures and Tables

**Table 1:** Patients’ and tumors’ characteristics.

Parameter	Value
Number of patients	45
Female Sex *n* (%)	45 (100)
Mean age	55.6 ± 12.5 years, range: 31.3–86.0
Patients with recurrent disease *n* (%)	11 (24.4)
Number of tumors	56
Mean tumor diameter	1.6 cm (±0.7 cm)
Mean tumor volume	2.3 mL (±2.4 mL)
Tumor size	
≤2 cm *n* (%)	46 (82.1)
>2 cm *n* (%)	10 (17.9)
Patients with metastatic disease	21 (46.6)
Axillary Lymph node metastases *n* (%)	18 (85.7)
Other organ metastases *n* (%)	3 (14.3)

**Table 2:** Ablation procedure.

Parameter	Value
Number of ablations	56
Mean ablation time	16.6 min
Initial Complete ablations *n* (%)	56 (100)
Mean ice ball diameter	
Ice ball 1 (length × width)	4.1 × 2.8 cm
Ice ball 2 (length × width)	4.5 × 3.2 cm
Mean ice ball size	4.3 × 3.0 cm
Mean ablation zone size 24 h-post:	
Volume	38.6 mL (±17.5 mL)
Diameter	5.3 cm (±1.4 cm)
Mean reduction ablation zone volume:	
3 months post-ablation	69.4% (±16.5%)
total	80.5% (±20.1%)
Complication-free ablations *n* (%)	56 (100)

**Table 3:** Patients’ Outcome after Cryoablation.

Parameter	Value
Local tumor progression *n* (%)	4 (8.9)
Intramammary distant recurrence *n* (%)	6 (13.3)
Extramammary tumor progression *n* (%)	7 (15.6)
Mean overall survival	4.13 years (95% CI: 3.7–4.5)
Mean overall progression-free survival	2.5 years (95% CI: 1.8–3.2)
Mean local progression-free survival	2.9 years (95% CI: 2.3–3.6)
6-month overall progression-free survival rate	85.4%
12-month overall progression-free survival rate	72.3%
6-month local progression-free survival rate	90%
12-month local progression-free survival rate	80%

## Data Availability

Data may be requested from the corresponding author. All requests will be checked according to privacy or ethical restrictions.
